# Homelessness Following Jail Exit Among Previously Housed Individuals

**DOI:** 10.1007/s11524-025-01016-4

**Published:** 2025-10-29

**Authors:** Emily E. Ager, Meghan M. Hewlett, Dallas Augustine, Eve Perry, Hemal K. Kanzaria, Kenneth Perez, Jacob Izenberg, Maria C. Raven

**Affiliations:** 1 Department of Emergency Medicine, University of California San Francisco, 521 Parnassus Avenue, San Francisco, CA 94143 USA; 2 Department of Justice Studies, San Jose State University, San Jose, CA USA; 3 Benioff Homelessness and Housing Initiative, University of California San Francisco, San Francisco, CA USA; 4 Phillip R. Lee Institute for Health Policy Studies, University of California San Francisco, San Francisco, CA USA; 5 Department of Psychiatry and Behavioral Sciences, University of California San Francisco, San Francisco, CA USA

**Keywords:** Homelessness, Incarceration, Jail

## Abstract

Incarceration is a recognized risk factor for homelessness. However, most research focuses on the relationship between homelessness and prison incarceration. Jail incarceration is more common compared to prison incarceration, but little data exists on its impact on housing. The objective of this study is to examine the occurrence of housing loss after jail incarceration among individuals without prior evidence of homelessness and the associated risk of reincarceration. In this retrospective cross-sectional study, we identified adults without evidence of homelessness who became unhoused within 6 months of jail incarceration. We compare pre-incarceration emergent and urgent health and social services utilization among housed and unhoused individuals, as well as the risk of reincarceration. Data are from the San Francisco (SF) Department of Public Health Coordinated Care Management System linked with SF City and County criminal justice data during fiscal years 2015–2018. We find that a quarter (25.1%) of individuals lost housing after jail incarceration, with a median incarceration length of 4 days in both the housed and unhoused groups. Compared to those without evidence of housing loss, more unhoused individuals had pre-incarceration substance use and mental health diagnoses and related service utilization. Unhoused individuals had 1.9 greater odds of reincarceration. In conclusion, we find that a significant number of individuals had evidence of housing loss after even a short jail incarceration; behavioral health diagnoses were more common among this group. Housing loss was associated with subsequent reincarceration. Given our findings, jail re-entry programs would benefit from incorporating housing assistance and housing loss mitigation strategies.

**Supplementary Information:**

The online version contains supplementary material available at 10.1007/s11524-025-01016-4.

## Introduction

Homelessness and incarceration are two interrelated crises. While homelessness is a risk factor for incarceration, incarceration itself is increasingly recognized as disruptive to housing stability and a risk factor for homelessness [1, 3]. U.S. adults with a history of prison incarceration have higher rates of housing and financial insecurity compared to those with no prior prison incarceration [4, 5]. These vulnerabilities are particularly pronounced immediately following prison release, when individuals are at highest risk of becoming homeless due to disruptions caused by the incarceration (e.g., missed rent, eviction, ruptured family ties, loss of employment) [1, 6].

Most research related to housing and incarceration focuses on individuals released from prison rather than from jail, leaving gaps in the literature regarding the relationship between jail and housing status. While 1.6 million individuals are incarcerated in U.S. federal and state prisons, there were 7.3 million U.S. jail incarcerations from July 2021 to June 2022 [5, 7]. Jail incarceration differs from prison incarceration in that the average jail incarceration duration is shorter—in 2022, individuals in U.S. jails spent an average of 32 days in custody before release, whereas the average time served by state prisoners released in 2018 was 2.7 years [7, 8]. Individuals in jail are often detained during a pretrial period, when they have not yet been convicted of a crime, and may remain in jail during this period due to an inability to afford bail, public safety concerns, or a perceived inability to participate in the court process [9]. Jail incarcerations also occur more frequently than incarceration in prison and present a unique set of problems for those who are already at an economic disadvantage [9]. Frequent, short-term incarcerations disrupt financial stability, employment, and social ties, all of which are needed to help individuals maintain housing and avoid interaction with the criminal justice system [11].

Research on the link between prison incarceration and homelessness often highlights the role of release, documenting difficulties with re-entering society during this time [3, 4]. For example, men recently released from prison were twice as likely to experience homelessness compared to those who were not recently in prison [4]. Release from prison is also associated with frequent moves and the need to live with other individuals in order to pay rent [4]. Similar relationships may hold true for jail incarceration. Those who are jail-incarcerated face significant economic hardship—prior to incarceration, their median annual income is 48% of the median income for non-incarcerated individuals of similar age and 79% of the median pre-incarceration income for those in state prisons [5, 12]. Low income also increases vulnerability to housing instability following jail release [5, 12]. While research has shown that many individuals cycle between homelessness and jail, less is known about individuals who enter jail housed, but exit into homelessness, and their risk of subsequent reincarceration [3, 13].

To our knowledge, no studies have looked directly at housing loss after jail incarceration among previously housed individuals. We address this gap by using a unique, integrated dataset to examine the occurrence of homelessness among individuals without evidence of recent homelessness prior to incarceration in jail. We compare individual-level factors between individuals with and without evidence of post-incarceration homelessness, including prior criminal justice system interactions, pre-incarceration emergent or urgent behavioral and physical healthcare utilization, and characteristics of the index incarceration. Finally, we estimate the risk of a repeat jail booking among unhoused individuals compared to those without evidence of homelessness.

## Methods

### Study Design and Setting

We conducted a retrospective cross-sectional study of adults in the City and County of San Francisco using data from the San Francisco Department of Public Health (SFDPH) Coordinated Care Management System (CCMS) registry linked with criminal justice system data from the San Francisco County District Attorney and Sheriff’s Office for fiscal years (FY) 2015–2018 (July 1, 2015–June 30, 2018). We chose to restrict our study period to the pre-Covid-19 period, as during this period, there were several County-wide changes that risked biasing our analysis, including a large decrease in the County jail population and a substantial increase in resources and support for unhoused individuals in San Francisco.

The construction of the CCMS has previously been comprehensively described [14, 17]. Briefly, the CCMS is a composite database implemented by the SFDPH that integrates information from multiple data sources on physical health, mental health, substance use, housing status, social service utilization, and criminal justice interactions among the high-risk and vulnerable population served by the SFDPH [14, 17]. These data are extracted by the SFDPH from several County agencies and from San Francisco’s primary Medicaid-managed care plan, the San Francisco Health Plan. Individuals have a record in the CCMS if they utilize urgent or emergent County services related to physical health, mental health, or substance use disorders (SUD), are reported as unhoused by a San Francisco County agency, or have contact with County jail health services. Most individuals in the CCMS are insured by Medi-Cal, California’s Medicaid program. Further details on the construction of the CCMS are available in the supplemental methods.

Episodes of homelessness are captured by the CCMS through both observed and reported events. First, homelessness status is observed if an individual accesses services recorded in CCMS that are only used by people experiencing homelessness. These include the city’s medical respite, shelter services, navigation centers, and stabilization rooms, as well as interactions with street-based homeless outreach teams. Homelessness is also observed based on data reported to the CCMS from the San Francisco Department of Homelessness and Supportive Housing, including the date of any completed assessments for Adult Coordinated Entry, the county’s system for permanent supportive housing prioritization. Second, homelessness is captured in the CCMS if it is self-reported during a health services encounter (e.g., a physical or behavioral health clinical encounter) at the time of intake or registration. Patients without observed or reported homelessness are classified as housed. Housing ascertainment in the CCMS has been previously described in detail [14, 15].

We linked the CCMS database with jail booking data from the City and County of San Francisco Sheriff’s Office and data from all cases brought forth for potential prosecution from the County District Attorney. Technical aspects of this linkage process have been described in detail [14]. In brief, the linkage process used supervised machine learning to create a common individual identifier across the three datasets from first names, last names, and dates of birth; additional details are available in the supplemental methods [14]. Of the 278,828 individuals across both data sources in the entire 10-year period of data linkage (FY 11–20), 30,755 individuals had contact with both the CCMS and criminal justice systems [14]. All data linkage, storage, and analysis were conducted using a secure, HIPAA compliant platform for sensitive data. Researchers trained and approved to work with Protected Health Information analyzed the data. We obtained approval for this study through the University of California San Francisco Institutional Review Board. Reporting complies with the STROBE guidelines [18].

### Study Population and Measures

We define the study population with the following criteria: at least 18 years of age, a San Francisco County jail incarceration related to new criminal activity (not a booking related to a prior offense) during FY 2015–2018, at least one service utilization record in the CCMS in the FY prior to the index booking date, and no CCMS record indicating homelessness during a system encounter in the 6 months prior to the index booking date. We excluded individuals with a San Francisco County jail incarceration in the 365 days preceding their index booking date to avoid including individuals already in a cycle of multiple incarcerations that are often related to a single offense, possibly causing housing instability. Our primary outcome is the cumulative incidence of homelessness (recorded evidence of homelessness in the CCMS) in the 6-month period following release from the index jail incarceration.

In the period prior to the index booking date, we examine individuals’ demographics (age, race, ethnicity, primary language, gender), prior urgent or emergent medical service utilization (emergency department visit, urgent care visit, inpatient hospital stay), prior urgent or emergent mental health service utilization (psychiatric emergency service visit, psychiatric urgent care visit, inpatient psychiatric hospital stay), mental health diagnoses, urgent or emergent substance use service utilization (medical detoxification stay, social detoxification stay, sobering center visit), substance use history, and history of homelessness (prior to the 6 months before the index booking date). Demographic information was acquired from the CCMS, where it is self-reported; if these variables were not available in CCMS records, they were ascertained from Sheriff’s Office records. We also describe criminal justice system interactions in San Francisco County prior to the index jail incarceration, the index jail incarceration length of stay, characteristics of the offense leading to the index jail incarceration, and a new jail booking (the process that occurs when an individual is arrested and taken to jail) or jail case (the legal proceedings that follow a booking) within 6 months of release from the index jail incarceration.

### Statistical Analysis

After defining our study population, we stratify individuals into two sub-groups: those without evidence of homelessness and those with a CCMS record for homelessness within 6 months after the index jail incarceration. We then use descriptive statistics to examine the characteristics of each sub-group by the variables listed above. We report frequencies for categorical variables and medians with interquartile range (IQR) for continuous variables. We conducted Pearson’s chi-squared tests for categorical data and Welch’s two sample *t*-tests for continuous data to examine for differences in the variables of interest by each sub-group. We examined the 6-month post-incarceration cumulative incidence (CI) of homelessness by quartiles of index incarceration length and the number of reincarceration events to understand the impact of these exposures in more detail on our primary outcome. Quartiles of index incarceration length were used to account for positive skew in this variable. We report CI ratios (CIR) for each quartile compared to the first quartile to represent the risk of homelessness among individuals exposed to a longer duration of incarceration or a greater number of reincarceration events.

Finally, we use a multiple variable logistic regression model to estimate the risk of reincarceration among individuals who became unhoused compared to those without evidence of homelessness after the index incarceration. This model included controls for age, race, ethnicity, gender, housing status prior to the index incarceration, previous county jail incarcerations, psychoses and substance use diagnoses, length of index incarceration, and most serious charge type (misdemeanor or felony) at the time of the index incarceration. We report odds ratios (OR) and 95% confidence intervals. We used R version 4.3 for all statistical analyses.

## Results

### Population Characteristics

**Fig. 1:**
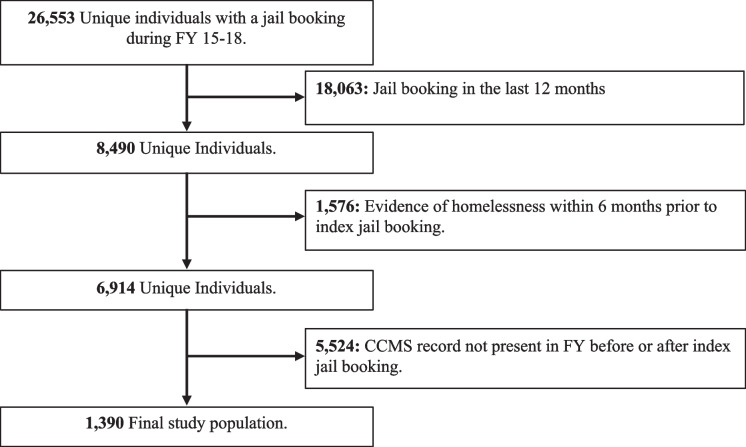
Study population selection flowchart. Abbreviations: *FY*, fiscal year

In total, 26,553 unique individuals had a jail booking during the study period; 18,063 individuals were excluded for having a jail booking in the preceding 12 months; 1390 individuals met the study criteria (Fig. 1). The median age was 38 (IQR 30–49) and the majority were male (76.4%). Overall, 48.6% of individuals were Black, 28.8% were white, 13.7% were Latine, 5.9% were Asian/Pacific Islander, and 2.9% were Native American, Multi-racial, or “Other” (categories collapsed due to small cell size).

Within 6 months after release from their index jail incarceration, 349 (25.1%) of the study population have a record for homelessness in the CCMS. Both groups have a similar median age at index booking (40 (IQR 32–49) vs. 38 (IQR 30–49) years, respectively) and gender distribution (male 75.1% vs. 76.8%, respectively) (Table 1). 

**Table 1:** Demographics and index incarceration characteristics among individuals with and without post-incarceration homelessness (FY 2015–2018)

	Homeless after jail exit		Not homeless after jail exit	
	Total *N* = 1390			
	No. (%) *n* = 349 (25.1%)		No. (%) *n* = 1041 (74.9%)	
**Demographics**				
Age, median, (IQR) (years)	40	(32, 39)	38	(30, 49)
Age, mean, (± SD) (years)	40.6	(11.1)	39.5	(12.1)
18–24	18	(5.2%)	120	(11.5%)
25–49	248	(71.1%)	680	(65.3%)
50 +	83	(23.8%)	241	(23.2%)
Gender				
Female	87	(24.9%)	241	(23.2%)
Male	262	(75.1%)	800	(76.8%)
Race/ethnicity				
Black	136	(39.0%)	539	(51.8%)
White	141	(40.4%)	260	(25.0%)
Latine	36	(10.3%)	155	(14.9%)
Asian/Pacific Islander	21	(6.0%)	61	(5.9%)
Native American/Other/Multi-racial^a^	15	(4.3%)	26	(2.5%)
**Index incarceration characteristics**				
Length of index incarceration (days)				
Median (IQR)	4	(2, 18)	4	(2, 8)
Mean (SD)	24.6	(58.0)	19.6	(69.7)
Range	-	1–643	-	1–1090
Most serious active charge type				
Felony	291	(83.4%)	860	(84.0%)
Misdemeanor	58	(16.6%)	164	(16.0%)

^a^Categories collapsed due to cell size less than 10. Numbers may not sum to 100% due to rounding error. *FY*, fiscal year; *IQR*, interquartile range; *SD*, standard deviation

### Index Jail Incarceration Characteristics

The median and mean length of the index incarceration were similar, with a median length of 4 days in both groups (IQR unhoused 2–18; IQR housed 2–8), mean length of 24.6 days (range 1–643) in the unhoused group, and mean length of 19.6 days (range 1–1090) in the group without evidence of homelessness. There were no significant differences in the most serious active charge type between the two groups; felonies account for 83.4% of charges in the unhoused group and 84.0% in the not-unhoused group; misdemeanors account for 16.6% of charges in the unhoused group and 16.0% in the not-unhoused group. See Table 1.

Among individuals in the first quartile of incarceration length (< 1.9 days), the CI of homelessness was 27.0% over the 6-month post-incarceration period. Among those in the second quartile (2.0–3.6 days), the CI was 18.1%, and the CIR compared to the first quartile was 0.67 (*p* = 0.005). Those in the third quartile (3.7–10.0 days) had a CI of homelessness of 23.6% and a CIR of 0.88% (*p* = 0.3). Individuals in the fourth quartile (≥ 10.1 days) had a CI of 31.7% and CIR of 1.17 compared to the first quartile (*p* = 0.2).

### Physical Health, Mental Health, and Substance Use History

Compared to individuals without evidence of homelessness, those with a post-incarceration homelessness episode had a higher pre-incarceration use of urgent or emergent mental health care services (23.5% vs. 15.9%; *p* < 0.001) and diagnosis of psychosis (22.3% vs. 13.7%; *p* < 0.001) in the year prior to the index booking (Table 2). Those who became homeless also had a higher pre-incarceration use of urgent or emergent substance use services (12.6% vs. 8.6%; *p* = 0.001) and substance use disorder diagnosis (46.1% vs. 36.2%; *p* < 0.001) in the year prior to the index booking. Utilization of urgent or emergent medical services was similar between the groups during this period (92.0% vs. 94.1%; *p* = 0.4). See Table 2.

**Table 2:** Health-related service utilization and diagnoses, pre-and post-incarceration criminal justice system interactions, and housing status among individuals with and without post-incarceration homelessness (FY 2015–2018)

	Homeless after jail exit No. (%) *n* = 349 (25.1%)		Not homeless after jail exit No. (%) *n* = 1041 (74.9%)		***p***-value
Urgent or emergent health-related service utilization					
Medical care					0.4
Prior FY	321	(92.0%)	980	(94.1%)	
2 + prior FYs	19	(5.4%)	40	(3.8)	
Never	-^a^	-^a^	21	(2.0%)	
Mental health care					< 0.001
Prior FY	82	(23.5%)	165	(15.9%)	
2 + prior FYs	65	(18.6%)	100	(9.6%)	
Never	202	(57.9%)	776	(74.5%)	
Substance use care					0.001
Prior FY	44	(12.6%)	90	(8.6%)	
2 + prior FYs	58	(16.6%)	117	(11.2%)	
Never	247	(70.8%)	834	(80.1%)	
Medical diagnoses					
Depression					0.10
Prior FY	49	(14.0%)	145	(13.9%)	
2 + prior FYs	52	(14.9%)	111	(10.7%)	
Never	248	(71.1%)	785	(75.4%)	
Psychosis					< 0.001
Prior FY	78	(22.3%)	143	(13.7%)	
2 + prior FYs	33	(9.5%)	51	(4.9%)	
Never	238	(68.2%)	847	(81.4%)	
Substance use					< 0.001
Prior FY	161	(46.1%)	377	(36.2%)	
2 + prior FYs	77	(22.1%)	168	(16.1%)	
Never	111	(31.8%)	496	(47.6%)	
Homelessness prior to index incarceration^b^					
Evidence of homelessness					< 0.001
Within 6–12 months	132	(37.8%)	187	(18.0%)	
> 12 months	128	(36.7%)	337	(32.4%)	
Never	89	(25.5%)	517	(49.7)	
Pre-incarceration criminal justice system interactions^b^					
Prior jail bookings	278	(79.7%)	804	(77.2%)	0.4
Prior cumulative days incarcerated					0.2
Median (IQR)	69	(6, 290)	43	(4, 259)	
Mean (SD)	186.0	(243.7)	164.9	(236.8)	
Range	-	0–1348	-	0–1498	
Post-incarceration criminal justice system interactions^b^					
Incarceration charge type					
Any charge	230	(65.9%)	514	(49.4%)	< 0.001
Any new charge	206	(59.0%)	451	(43.3%)	< 0.001
Any existing charge	98	(28.1%)	159	(15.3%)	< 0.001
New felony	155	(44.4%)	335	(32.2%)	< 0.001
New misdemeanor	106	(30.4%)	223	(21.4%)	< 0.001
Number of reincarcerations					< 0.001
Median (IQR)	1	(1, 2)	0	(0, 2)	
Mean (SD)	1.7	(2.0)	1.0	(1.5)	
Range	-	0–20	-	0–12	
Cumulative days reincarcerated					0.4
Median (IQR)	22	(5, 83)	13	(2, 71)	
Mean (SD)	61.2	(96.9)	55.1	(97.2)	
Range	-	0–774	-	0–703	

^a^Not reported given cell size less than 10

^b^Recorded in the city and county of San Francisco. Numbers may not sum to 100% due to rounding error

*FY*, fiscal year; *IQR*, interquartile range; *SD*, standard deviation

### Housing and Incarceration History

Those who became unhoused had more homelessness records prior to the 6 months before the index incarceration compared to individuals without evidence of homelessness (Table 2). The two groups had similar proportions of jail bookings prior to 1 year before their index incarceration (79.7% vs. 77.2%; *p* = 0.4). The median number of cumulative days incarcerated prior to the index jail incarceration was similar: 69 days in the unhoused group (IQR 6–290) and 43 days in the not-unhoused group (IQR 4–259); *p* = 0.4.

### Post-Incarceration Criminal Justice Interactions

Compared to individuals without evidence of homelessness, a higher proportion of those who became unhoused were reincarcerated in jail for any charge (65.9% vs. 49.4%; *p* < 0.001), any new charge (59.0% vs.43.3%; *p* < 0.001), or any existing charge (28.1% vs. 15.3%; *p* < 0.001) within 1 year of release. The unhoused group had a higher median number of reincarceration events compared to the not-unhoused group (1 (IQR 0–2) vs. 0 (IQR 0–2); *p* < 0.001); there was no significant difference in the median or mean number of cumulative days reincarcerated between the two groups (*p* = 0.4) (Table 2). Compared to housed individuals, a higher proportion of unhoused individuals were reincarcerated for a new felony charge (44.4% vs. 32.2%; *p* < 0.001) or a new misdemeanor charge (30.4% vs. 21.4%; *p* < 0.001). See Table 2.

Increasing reincarceration events was associated with a higher CIR of homelessness. Individuals with no subsequent reincarcerations had a CI of homelessness of 18.4% within 6 months of the index incarceration. Those with one reincarceration had a CI of homelessness of 24.5% and a CIR of 1.33 compared to those with no reincarcerations (*p* = 0.028). Individuals with 2 or > 2 reincarceration events had a CIR of homelessness of 1.86 (*p* < 0.001) and 2.02 (*p* < 0.001), respectively, compared to those with no reincarcerations. See Table 3

**Table 3:** Cumulative incidence ratio of post-index incarceration homelessness by number of post-index incarceration reincarceration events

Number of reincarcerations	N	6-month post-incarceration CI of homelessness	CI ratio^a^	Ratio *p*-value
0	646	18.4%	1	-
1	323	24.5%	1.33	0.028
2	193	34.2%	1.86	< 0.001
> 2	228	37.3%	2.02	< 0.001

^a^Calculated relative to group with 0 reincarcerations

Compared to those without a homelessness episode, individuals who became unhoused after their index incarceration had 1.86 times greater adjusted odds of having any repeat jail booking within 1 year after the index incarceration (95% CI 1.41–2.45; *p* < 0.001). Unhoused individuals had 1.88 times greater adjusted odds of having a jail booking for a new criminal case within 1 year of release from the index incarceration (95% CI 1.44–2.46; *p* < 0.001).

## Discussion

To our knowledge, this is the first exploration of the association between jail incarceration and housing loss among previously housed individuals. We found that a quarter of individuals had a record for a homelessness episode within 6 months after release from jail, despite this group having the same median incarceration duration as those without evidence of homelessness(4 days). We also found that the CI of homelessness did not significantly increase as the incarceration duration increased. While housing instability may be more expected after a long period of incarceration due to missed rent, loss of employment, or other disruptions, we found a high occurrence of homelessness even after a short duration of incarceration.

Our findings reflect the underlying social vulnerabilities among our study population. For example, a third experienced homelessness prior to the year before their index jail incarceration and had higher diagnoses of psychosis (15.9%) and substance use (38.7%) compared to the general U.S. population [19, 20]. These characteristics reflect the national jail populus, who is more likely to have serious mental illness, underlying health conditions, and SUD compared to non-incarcerated individuals [21, 22]. The high prevalence of behavioral and physical health conditions among incarcerated individuals reflects inconsistent and fragmented access to mental health and substance use treatment services in the U.S.A. [23]. Due to inadequate levels of community-based physical and behavioral health services, law enforcement and the carceral system are increasingly used to respond to medically and socially marginalized individuals [3, 9, 24].

Though our study population had an overall high service utilization for mental health and substance use care in San Francisco County, those who became homeless had greater pre-incarceration use of these services. This may be a marker of increased physical and behavioral health disease severity and need for social services [25]. Specifically, substance use can increase the risk of interactions with law enforcement and low-level offenses for substance use and related behaviors [21]. This can result in an increased risk of jail incarceration, further interfering with receiving SUD treatment in a therapeutic environment. While behavioral health treatment services are readily available through SFDPH Jail Health Services, the jail environment may increase the risk of instability at the time of release for those with mental illness or SUD compared to those without them [26, 27]. Incarcerated individuals with SUD may also experience withdrawal or be unable to receive appropriate treatment, such as medications for opioid use disorder (MOUD). While MOUD is available in San Francisco County jails, nationally, these medications are not universally available in jail and their discontinuation can increase the risk of opioid-related overdose after release [22].

Additionally, compared to those without evidence of homelessness, we find that individuals who became unhoused had an almost two times greater odds of having a repeat jail booking within a year after release; in fact, most unhoused individuals (65.9%) were reincarcerated within 6 months. We also report a dose-dependent relationship between reincarceration events and homelessness, suggesting a single jail incarceration can be associated with a cycle of homelessness and incarceration. This is consistent with prior research showing an increased prevalence of homelessness among those in jail compared to the general population—an analysis of city jail bookings in Atlanta found that 12.5% of individuals in jail were unhoused compared to 0.4% of the city’s entire population [24]. Prior studies also find that medically and economically vulnerable individuals are more likely to have repeat jail incarcerations [21].

Entering a cycle of incarceration and homelessness can occur by several mechanisms. First, an incarceration history creates barriers to housing due to policies requiring criminal background checks as part of the public housing application process [28]. Next, especially in areas where unsheltered homelessness predominates such as California, homelessness can result in increased law enforcement contact and charges for lower-level offenses, such as trespassing [24, 29]. Several states, including California in 2014, have reclassified minor misdemeanors into non-jailable offenses or infractions to avoid incarceration [30]. Unhoused individuals may also struggle to attend court hearings due to transportation or other barriers. This can result in a “bench warrant” for failure to appear in court and possibly a jail booking [24].

Finally, the 2024 U.S. Supreme Court decision in *City of Grants Pass v. Johnson* allowed for the criminalization of sleeping outdoors on public property [31]. Implementing such policies may further increase the risk of law enforcement contact and subsequent jail incarceration among unhoused individuals. Combined, such policies create conditions where even a brief interaction with the criminal justice system can result in a cycle of homelessness, poverty, and incarceration.

There are several public policy changes that may mitigate the risks individuals face from cycles of incarceration and homelessness. First, every effort should be made to limit jailing those with low-level, nonviolent offenses. Diversion programs, such as the Behavioral Health Court in the County of San Francisco established in November 2002, aim to reduce reincarceration among individuals with serious mental illness by linking them with wraparound community services [32]. The expansion of such programs and continued support and resources for existing programs can help individuals avoid incarceration. Additionally, housing and residential treatment facilities tailored to the needs of individuals with previous criminal justice involvement may provide more reliable and stable options after release from incarceration. Further, the cash bail system, still widely in use in the U.S.A., disproportionately impacts poor individuals and people of color. The reduction or removal of money bail can create a more equitable criminal justice system [33].

Individuals incarcerated in jail may experience systemic barriers to successful community re-entry. Our findings support previous studies describing the vulnerable health and economic status among individuals in jail and highlight the need for robust re-entry services, even after a brief incarceration [9, 21]. Federal law previously barred the use of federal Medicaid funds for services for incarcerated individuals. However, in 2023, as part of the California Advancing and Innovating Medi-Cal (CalAIM) initiative, California became the first state to receive federal approval to use the state’s Medicaid health care program to cover certain services in the 90 days before youth and adults are released from county jails, state prisons, and youth correctional facilities [34]. This initiative aims to ensure that all eligible individuals leave prison or jail enrolled in Medi-Cal and connected to appropriate services, such as case management and physical and behavioral health care. Our findings would also support the need to include services that can prevent homelessness.

### Limitations

A limitation to our study is that our analysis included only records available in the CCMS. Given the structure of CCMS data, which is linked to service utilization in the City and County of San Francisco, it is possible that the presence of homelessness was undercounted both before and after the jail incarceration. Additionally, it is possible that individuals with a medical or behavioral health-related hospitalization may have otherwise been unhoused, leading to an underestimation of the prevalence of homelessness during the study period. However, the CCMS has been utilized in many previous studies that demonstrate its validity and reliability as a data source for research purposes, including studies that aim to categorize housing status [14, 15, 17]. Further, this linked dataset provides a unique ability to capture patient-level information related to behavioral health, physical health, social service utilization, and criminal justice system interactions that is not otherwise available. Though we found an association between post-index homelessness and reincarceration, because we did not use event-level data in the period following the index incarceration and do not know the disposition of individuals immediately upon release from jail, we cannot definitively say which event occurred first in the post-incarceration period—reincarceration or homelessness. Regardless, based on the dose-response relationship we find between reincarceration and homelessness and existing literature on the topic, there is a strong association between the two events.

Finally, our analysis is limited to examining the occurrence of homelessness in the 6 months after release from jail. Though most research on the relationship between incarceration and housing stability focuses on the immediate post-incarceration period, residual social, economic, and medical instability, and the reinforcement of these factors on one another among previously incarcerated individuals may increase the risk of housing loss over the life course.

## Conclusion

This study provides the first exploration of the association between jail incarceration and housing loss among previously housed individuals. We use a unique dataset linking emergent or urgent health and social services utilization with county-level criminal justice system data. We find that a quarter of our study population had evidence of housing loss within 6 months of exiting jail. Pre-incarceration behavioral health diagnoses and related health services utilization were higher among this group. We also find that individuals who became unhoused had almost two times greater odds of a repeat jail booking. Given our findings, jail re-entry programs would benefit from incorporating or strengthening existing housing assistance and housing loss mitigation strategies.

## Supplementary Information

Below is the link to the electronic supplementary material.

ESM1 (DOCX 26.5 KB)

## Data Availability

The Coordinated Care Management System data and the criminal justice data from the San Francisco County District Attorney and Sheriff's Office used in this study is not publicly available.
