# Supplementary material for: Homelessness Following Jail Exit Among Previously Housed Individuals
Source: J Urban Health. 2025 Oct 29;102(5):977–88. doi: 10.1007/s11524-025-01016-4 (PMC12669472; doi:10.1007/s11524-025-01016-4)
Supplement: Supplementary file 1 — (DOCX 26.5 KB) [file 11524_2025_1016_MOESM1_ESM.docx]

**Supplemental Methods**

**Description of the San Francisco Department of Public Health Coordinated Care Management System (CCMS)**

The Coordinated Care Management System (CCMS) is an integrated data repository implemented by the San Francisco Department of Public Health (SFDPH) that links health and social service utilization records. The CCMS integrates information from many data sources across San Francisco County in order to monitor the health and social services utilization among the complex, high risk, and vulnerable population served by the SFDPH. The CCMS links information related to service utilization for emergent or urgent physical health services, urgent or emergent mental health services, and urgent or emergent substance use services among individuals served by the SFDPH. Physical and behavioral health services captured in the CCMS database are shown in Supplemental Table 1. Additionally, the CCMS includes data on social services utilization such as housing, shelter use, and jail health services encounters. These data are provided to the CCMS by multiple county agencies and San Francisco County’s primary Medicaid managed care plan, the San Francisco Health Plan. Additional details regarding the services and data included in the CCMS are available in Appendix Exhibit A2 in Kanzaria et al. 2019.

A comprehensive explanation of the development and maintenance of the CCMS has been described in prior literature (Kanzaria et al., 2019). Briefly, the SFDPH matches and integrates patient-level data within the CCMS using individuals’ last name, first name, date of birth, and social security number using computer probability algorithms and manual confirmation. Records are continuously updated.

| Supplemental Table 1. Catalog of Urgent and Emergent Services Captured by the San Francisco Department of Public Health’s Coordinated Care Management System. | | |
| --- | --- | --- |
| **System** | **Urgent/Emergent Service** | **Unit of Measure** |
| **Physical Health System** | Emergency Department | Visit |
|  | Hospital Medical Inpatient | Stay |
|  | Urgent Care Clinic | Visit |
| **Mental Health System** | Psychiatric Emergency Services | Visit |
|  | Hospital Psychiatric Inpatient | Stay |
|  | Psychiatric Urgent Care Clinic | Visit |
| **Substance Use Disorder System** | Medical Detoxification | Stay |
|  | Social Detoxification | Stay |
|  | Emergency Department | Visit |

**Record Creation in the CCMS**

A record is created in the CCMS for any individual with utilization of the urgent or emergent physical or behavioral health services included in Supplemental Table 1. Records are also created for individuals observed or reported to be unhoused by a healthcare or social services worker (additional details on housing ascertainment in the CCMS are provided below), as well as those who use county behavioral health, housing, or jail services. Records are matched with historical data at the patient level, then merged and integrated into the CCMS.

**Housing Ascertainment in the CCMS**

Episodes of homelessness are captured by the CCMS through both observed and reported events. First, homelessness status is observed if an individual accesses services recorded in CCMS that are only used by people experiencing homelessness. These include the city’s medical respite, shelter services, navigation centers and stabilization rooms, as well as interactions with street-based homeless outreach teams. Homelessness is also observed based on data reported to the CCMS from the San Francisco Department of Homelessness and Supportive Housing, including the date of any completed assessments for Adult Coordinated Entry, the county’s system for permanent supportive housing prioritization. Second, homelessness is captured in the CCMS if it is self-reported during a health services encounter (e.g. a physical or behavioral health clinical encounter) at the time of intake or registration. Patients without observed or reported homelessness are classified as housed.

**Dataset Linkage**

We linked the CCMS database with jail booking data from the City and County of San Francisco Sheriff’s Office and data from all cases brought forth for potential prosecution from the County District Attorney. Records were linked using first names, last names, and dates of birth to create a common individual identifier. A HIPAA-compliant platform was used for all data linkage, storage, and analysis. Researchers trained and approved to work with Protected Health Information analyzed the data.

Comprehensive technical details on the data linkage process are available in Appendix A.4 of “[Signals of Distress: High Utilization of Criminal Legal and Urgent and Emergent Health Services in San Francisco](https://capolicylab.org/wp-content/uploads/2022/10/Signals-of-Distress.-High-Utilization-in-San-Francisco.pdf).”

**Additional references:**

- Decker H, Evans J, Squire DG, Colom S, Perez K, Raven M, Plevin R, Kanzaria HK, Stey A. Timeliness of injury care and housing status. Injury. 2025 Jun 17:112531. doi: 10.1016/j.injury.2025.112531. Epub ahead of print. PMID: 40592661.
- Decker H, Evans J, Squire DG, Colom S, Perez K, Raven M, Plevin R, Kanzaria HK, Stey A. Housing Status and Longitudinal Care Patterns After Injury. Ann Emerg Med. 2025 Aug;86(2):158-168. doi: 10.1016/j.annemergmed.2025.03.024. Epub 2025 May 7. PMID: 40332060.
- Decker H, Colom S, Evans JL, Graham-Squire D, Perez K, Kushel M, Wick E, Raven MC, Kanzaria HK. Association of housing status and cancer diagnosis, care coordination and outcomes in a public hospital: a retrospective cohort study. BMJ Open. 2024 Sep 12;14(9):e088303. doi: 10.1136/bmjopen-2024-088303. PMID: 39266319; PMCID: PMC11404260.
- Decker H, Colom S, Graham-Squire D, Wick E, Kushel MB, Raven M, Kanzaria HK. Housing Status and Acute Care Use After Cancer Diagnosis. JAMA Netw Open. 2024 Jul 1;7(7):e2419657. doi: 10.1001/jamanetworkopen.2024.19657. PMID: 38954418; PMCID: PMC11220561.
- Eswaran V, Raven MC, Wang RC, Cawley C, Izenberg JM, Kanzaria HK. Understanding the association between frequent emergency department use and jail incarceration: A cross-sectional analysis. Acad Emerg Med. 2022 May;29(5):606-614. doi: 10.1111/acem.14437. Epub 2022 Feb 10. PMID: 35064709; PMCID: PMC10231420.
- Kanzaria HK, Niedzwiecki M, Cawley CL, et al. Frequent Emergency Department Users: Focusing Solely On Medical Utilization Misses The Whole Person. Health Aff (Millwood). 2019;38(11):1866-1875. doi:10.1377/hlthaff.2019.00082
- Cawley C, Raven MC, Martinez MX, Niedzwiecki M, Kushel MB, Kanzaria HK. Understanding the 100 highest users of health and social services in San Francisco. Acad Emerg Med. 2021;28(9):1077-1080. doi:10.1111/acem.14299
- Hewlett MM, Raven MC, Graham-Squire D, et al. Cluster Analysis of the Highest Users of Medical, Behavioral Health, and Social Services in San Francisco. J Gen Intern Med. 2023;38(5):1143-1151. doi:10.1007/s11606-022-07873-y
